# Quantitative and Qualitative HPLC Analysis of Mycosporine-Like Amino Acids Extracted in Distilled Water for Cosmetical Uses in Four Rhodophyta

**DOI:** 10.3390/md18010027

**Published:** 2019-12-28

**Authors:** Patricia Chaves-Peña, Francisca de la Coba, Felix L. Figueroa, Nathalie Korbee

**Affiliations:** 1 Department of Ecology and Geology, Faculty of Sciences, University of Malaga, Institute of Blue Biotechnology and Development (IBYDA), Campus Universitario de Teatinos s/n, E-29071 Malaga, Spain; patricia.ch@uma.es (P.C.-P.); Felix_lopez@uma.es (F.L.F.); 2 Photobiology Laboratory, Central Service for Research Support (SCAI), University of Malaga, Campus Universitario de Teatinos s/n, E-29071 Malaga, Spain; pdlacoba@uma.es

**Keywords:** HPLC column, mycosporine-like amino acids, photoprotection, re-dissolution solvent, Rhodophyta

## Abstract

Mycosporine-like amino acids (MAAs) have gained considerable attention as highly active photoprotective candidates for human sunscreens. However, more studies are necessary to evaluate the extraction efficiencies of these metabolites in cosmetic compatible solvents, as well as, their subsequent HPLC analysis. In the present study, MAA extraction using distilled water and 20% aqueous methanol in four Rhodophyta was investigated. Different re-dissolution solvents and a C8 and C18 columns were tested for the HPLC analysis. Porphyra-334, shinorine, palythine, palythine-serine, asterina-330, and palythinol were identified by HPLC/ESI-MS. The separation of these MAAs were improved employing the C8-column, and using methanol as re-dissolution solvent. Regarding total MAAs concentrations, no differences between the two solvents were found. The highest MAA amounts were observed injecting them directly in the HPLC. According to these results, distilled water could be an excellent extraction solvent for MAAs. Nevertheless, the re-dissolution in pure methanol after dryness would be the best option for the qualitative analysis of the most common MAAs in these red algae. Our results entail important implications regarding the use of red macroalgae as promising candidates as environment-friendly sources of natural sunscreens.

## 1. Introduction

Solar radiation exposes the intertidal marine macroalgae to elevated irradiances of UV-B (280–315 nm) and UV-A (315–400 nm). In fact, depletion of the ozone layer in the stratosphere during the past two decades has intensified the levels of solar UV-B radiation, which has reached unexpected levels that can be harmful for many biological processes [1,2,3]. The UV radiation can produce several detrimental effects on biologically important molecules such as lipids, proteins, or DNA [4,5,6].

The marine organisms that are exposed to UV radiation can accumulate natural UV-absorbing compounds [7]. Among others, mycosporine-like amino acids (MAAs) [8,9] have drawn special attention as molecules act as antioxidants and provide photoprotection [10,11]. The main producers of these molecules in the marine coast are the red algae [12,13,14].

MAAs are a family of intracellular compounds of low molecular weight (<400 Da), they are distributed in a wide range of marine organisms, are water-soluble, and have high molar extinction coefficients (ε = 28,100–50,000 L·mol^−1^·cm^−1^). They are secondary metabolites capable of absorbing UV radiation with the maximum absorbance between 310 and 365 nm [15,16,17]. MAAs have a general structure that consist of cyclohexenone or cyclohexenimine chromophores conjugated with one or two amino acids that are responsible of UV absorption [18]. The high photostability of MAAs over a wide range of temperature and pH, together with their antioxidant properties, make them promising metabolites in the biotechnology industry. These compounds are recognized as strong UV-absorbing molecules that can be used as an active ingredient in sun-care cosmetic products, therefore could be a potential supplement of chemical filters just used in sunscreens, also due to the fact that they could satisfy consumers who want the incorporation of natural ingredients. Additionally, MAAs could be effective against actinic erythema, but can also protect the humans against other biological effects such as immune suppression or photo-oxidative damage [11,19,20,21,22,23,24,25]. So, MAAs are promising functional ingredients used for novel cosmeceuticals (cosmetic products with health benefits). In fact, MAAs have already been commercialized as Helioguard®365. This cosmetic reagent contains the MAAs, shinorine and porphyra-334, extracted from the red alga *Porphyra umbilicalis* and has been successfully commercialized as a natural and safe sunscreen compound. Nowadays, the MAA extraction protocols differ in type of solvents, temperature, and extraction times. The MAA extraction efficiencies and concentrations are affected by these conditions [26,27,28,29]. A variety of extraction and separation methods for MAAs have been developed and tested on a broad range of organisms [28]. Ethanol and methanol from 20% to 80% were performed in the extraction of MAAs. However, the potential use of MAAs as sunscreen and antioxidant additives in cosmeceutical products implies the isolation of MAAs using aqueous solution preferably, discarding some organic solvents like methanol due to its toxicity. 

On the other hand, different chromatographic techniques have been used for isolation, identification, and quantification of MAAs. The most common method is reverse-phase high-performance liquid chromatography (HPLC), using monomeric octylsilicaC8 column [29] as well as low silanol-free group octadecylsilicaC18 column [16,30]. A mixture of distilled water:methanol with a low percentage of acetic acid run isocratically is the most common eluent.

Karsten et al. [28] were the first to evaluate the effect of re-dissolution solvents (100% methanol, distilled water, and HPLC eluent), after dryness, on the MAA extraction efficiency using different HPLC columns (Synergi C18, Sphereclone C8, and Luna C8) on a red and a green alga. However, to our knowledge, the extraction efficiency of distilled water as solvent has not been studied. So, an ideal method for MAA extraction and subsequent characterization is still an unsolved problem. The aim of the present study was to investigate the effect of two different extraction solvents and different post-extraction procedures, in the isolation, identification, and quantification of MAAs by HPLC, using two columns (C8 and C18). These procedures were tested in four Rhodophyta: *Agarophyton vermiculophyllum*, *Crassiphycus corneus*, *Gracilariopsis longissima* and *Pyropia leucosticta*. The most common used in MAA extraction, 20% methanol, and the most appropriated for the cosmetic industry, distilled water. For each, different post-extraction protocols were tested: Direct sample analysis by HPLC or evaporate the sample to dryness and then use different re-dissolving solvents (distilled water, HPLC eluent, and 100% methanol) before injection in HPLC. Additionally, for each protocol, two of the most used HPLC columns (C8 and C18) were tested.

## 2. Results

**Figure 1:**
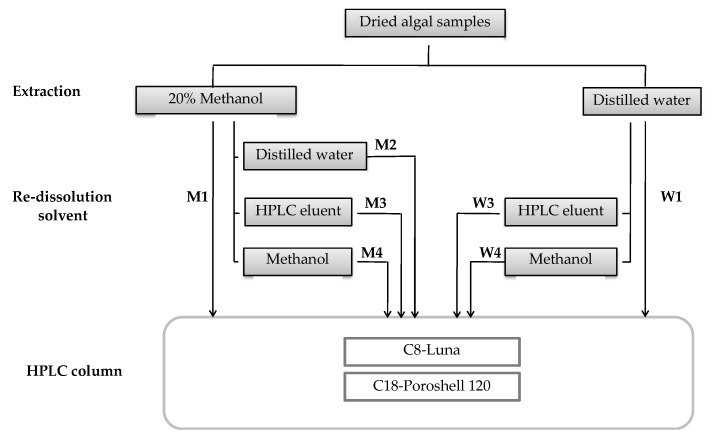
Extraction and re-dissolution protocols used to analyze mycosporine-like amino acids (MAAs); 20% aqueous methanol was used as extraction solvent in M1, M2, M3, and M4. Distilled water was utilized as extraction solvent in W1, W2, and W3. Number 1 indicates HPLC analysis done without re-dissolution. Number 2: Re-dissolution in distilled water. Number 3: Re-dissolution in HPLC eluent. Number 4: Re-dissolution in pure methanol.

In our study, we tested seven different methodological procedures for the extraction of MAAs and subsequent HPLC analysis in four Rhodophytes: *Agarophyton vermiculophyllum*, *Crassiphycus corneus*, *Gracilariopsis longissima,* and *Pyropia leucosticta*. Figure 1 illustrates those protocols. For each species, we tested two extraction solvents, 20% aqueous methanol and 100% distilled water. Some algal extracts were directly analyzed by HPLC, while others were dried off and subsequently re-dissolved in distilled water, in HPLC eluent (1.5% methanol plus 0.15% of acetic acid) and in pure methanol before being injected in HPLC. We did not consider the extraction and re-dissolution in distilled water procedure.

**Figure 2:**
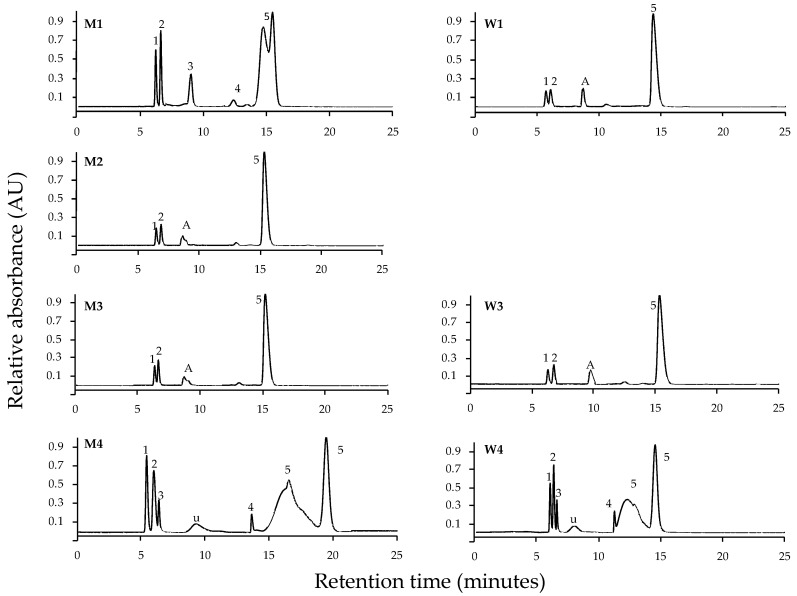
HPLC normalized chromatograms of MAAs identified in *Pyropia leucosticta* by seven methodological protocols using the Luna-C8 column. The code of protocol is indicated based on extraction and re-dissolution solvents used (see Figure 1 legend). Numbers indicate: 1 (palythine), 2 (asterina-330), 3 (palythinol), 4 (shinorine), 5 (porphyra-334), u (unidentifiable peak), and A (mixture of palythinol and shinorine).

**Figure 3:**
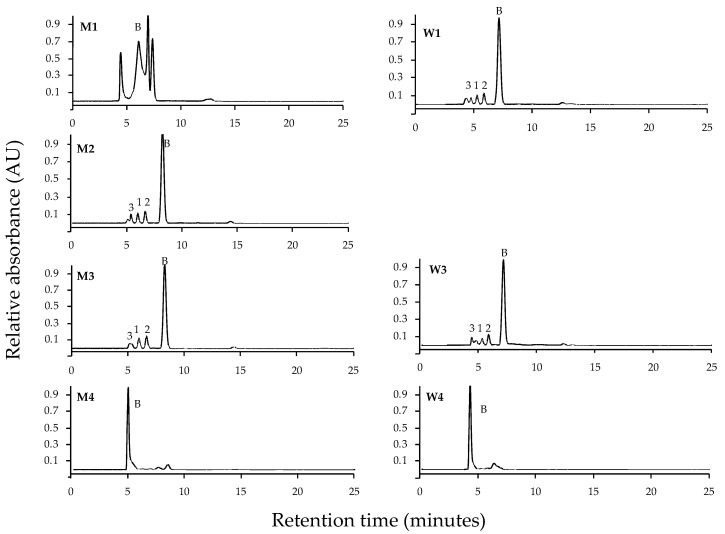
HPLC normalized chromatograms of MAAs identified of *Pyropia leucosticta* by seven methodological protocols using the Infinity Lab Poroshell 120 C18 column. The code of protocol is indicated based on extraction and re-dissolution solvents used (see Figure 1 legend). Numbers indicate: 1 (palythine), 2 (asterina-330), 3 (palythinol), and B (mixed unidentifiable peaks).

Chromatograms of *P. leucosticta* have been selected using C8 column (Figure 2) and C18 column (Figure 3). Chromatograms for the other species are shown in Figures S5–S10 (Supplementary Materials). In general, the use of the C18 column resulted in a deficient MAA separation (Figure 3). In three of the seven procedures, we could not identify different MAAs, so we observed a few mixed unidentifiable peaks. We had only been able to identify different MAAs using HPLC eluent or distilled water as a re-dissolution solvent, regardless of extraction solvent. The most used method for MAA analysis based on extract with 20% methanol and re-dissolute in 100% methanol (M4 protocol) resulted in a mix of MAAs using the C18 column. Regarding the MAA analysis using the C8 column, all protocols led to the separation of different MAAs. In fact, the protocols in which an improved MAA separation were M1, M4, and W4 for C8 column, while the separation was more efficient under M2, M3, W1, and W3 procedures for C18 column. However, none of them could separate all MAAs. According to these results, the incoming data presented in this work have been obtained applying the different procedures using the C8 column. 

The qualitative distribution of MAAs for each species was determined by ESI-MS and up to six different MAAs were identified in the analyzed samples: Porphyra-334, shinorine, palythine, asterina-330, palythinol, and palythine-serine (Table 1). Also, the relative proportions expressed in percentage were calculated in each alga according to the total concentrations obtained applying the procedures M4 and W4 (Table 1). Porphyra-334, shinorine, and asterina-330 were common in all studied species. Palythine and porphyra-334 were the dominants MAAs in *A. vermiculophyllum,* shinorine in *C. corneus,* palythinol in *G. longissima,* and porphyra-334 in *P. leucosticta*. Asterina-330 was the minority MAAs in all studied species except *P. leucosticta*, and palythine-serine was only present in *C. corneus.* Palythinol was only identified in three of these species: *G. longissima, C. corneus,* and *P. leucosticta*.

In addition, the flavonoid myricetin was identified by ESI-MS in *A. vermiculophyllum,* but no chromatographic peak was found associated to this molecule in any protocols or columns.

Using the C8 column, all MAAs described by ESI-MS were detected in *A. vermylophyllum* under all procedures. In *P. leucosticta,* only the M1, M4, and W4 protocols allowed a good identification (Table 2). In *C. corneus* and *G. longissima,* the protocols M4 and W4 offered similar results, i.e., only extraction in distilled water or 20% aqueous methanol and subsequent re-dissolution in 100% methanol must be accepted for the qualitative MAAs analysis. According to the obtained data, M4 and W4 would be the most appropriate procedures for the qualitative HPLC analysis of the following MAAs: Porphyra-334, shinorine, palythine, asterina-330, palythinol, and palythine-serine.

**Figure 4:**
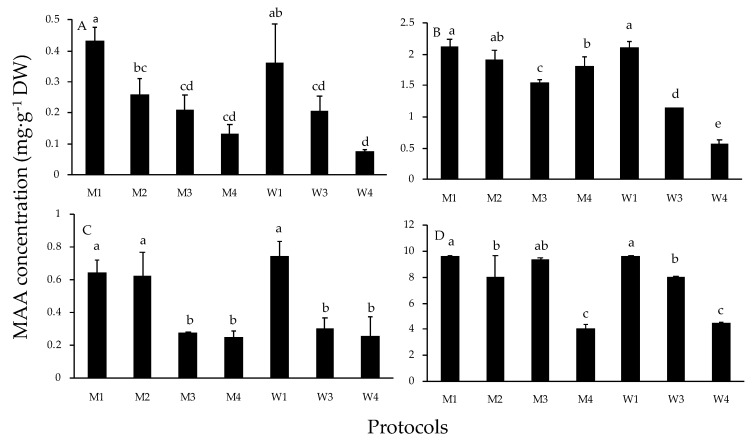
Concentration of total MAAs (mg g^−1^ DW) of each species obtained by seven different protocols. The code of protocol is indicated based on extraction and re-dissolution solvents used (see Figure 1 legend). (**A**) *Agarophyton vermiculophyllum*. (**B**) *Crassiphycus corneus*. (**C**) *Gracilariopsis longissima*. (**D**) *Pyropia leucosticta.* Different letters indicate significant differences among MAA concentrations for each species.

In general, total MAA concentrations did not differ between C18 and C8 columns (data not shown for C18). The concentration of MAAs calculated depended on the procedure in the four species (Figure 4). For C8 column, the highest MAA concentrations were observed in M1 and W1 procedures in the four Rhodophytes (*p* < 0.05). And no significant differences were found using distilled water or 20% aqueous methanol as extraction solvents in any species (Figure 4). The average value of MAA concentration for M1 and W1 procedures was 9.6 ± 0.04 mg g^−1^ DW in *P. leucosticta*, followed by *C. corneus* (2.11 ± 0.1 mg g^−1^ DW), *G. longissima* (0.69 ± 0.09 mg g^−1^ DW), and *A. vermiculophyllum* (0.4 ± 0.09 mg g^−1^DW). In general, M4 and W4 protocols offered the lowest MAA concentrations (*p* < 0.05) in the most studied algae. The average value of MAA concentration for these two procedures was 4.3 ± 0.26 mg g^−1^ DW in *P. leucosticta*, 1.19 ± 0.68 mg g^−1^ DW in *C. corneus*, 0.26 ± 0.07 mg g^−1^DW in *G. longissima*, and 0.1 ± 0.04 mg g^−1^ DW in *A. vermiculophyllum*. We found a tendency of progressive increases in MAA concentration as the percentage of methanol in the re-dissolution solvent decreased (Figure 4).

In the Table 3, the strengths and weaknesses of each protocol used in this work are summarized.

## 3. Discussion and Conclusions

It is well known that MAAs have high bioactive activities, therefore, there is a high interest to include MAAs in cosmetic formulations [20]. The potential uses of MAAs as sunscreen and antioxidant additives in cosmeceutical products implies the use of extraction solvents with no certified toxic effects and that allow the preservation of the physico-chemical and absorption properties of these molecules. MAAs have high solubility in aqueous solution; therefore, distilled water could be a good solvent in terms of extraction efficiency.

In order to study the influence of distilled water, as a solvent of extraction in MAA analysis, four Rhodophytes were selected based on their medium-high MAA contents and their different MAA profiles [17,31,32]: *Agarophyton vermiculophyllum*, *Crassiphycus corneus*, *Gracilariopsis longissima,* and *Pyropia leucosticta.* We selected different procedures and two HPLC columns that are commonly used for MAA analysis in order to elucidate which procedure would be the best option for the isolation, identification, and quantification of MAAs for cosmetic purposes. 

Different solvents had been used for MAA extraction, the most common are ethanol and methanol from 20% to 80% [14,17,30,33,34]. However, other solvents such as 2-octyl dodecanol, octyldodecyl ester of l-pyrrolidone carboxylic acid (OEL-PCA) (cosmetic emollient and moisturizer), and ethyl acetate revealed that UV absorption spectra of the extracts, number of elution chromatographic peaks, and their maxima absorption were affected by the nature of the solvents, being the extract in OEL-PCA, which showed the poorest UV absorption, and only palythenic acid could be identified in these extracts [35]. In our study, we compared the extraction in 20% aqueous methanol and in distilled water and no significant differences between them in terms of total MAA concentrations were found when these extracts were injected directly into HPLC. Furthermore, under this treatment, no hypsochromic shifts in UV-absorption maxima of the identified MAAs were observed.

Regarding qualitative and quantitative analysis of MAAs, HPLC-MS is the most used technique nowadays [36]. However, other techniques had been used such as capillary electrophoresis [33]. The most common columns used to separate and characterize MAAs by reverse-phase high-performance liquid chromatography (HPLC) are a monomeric octylsilica C8 column [29] and a low silanol-free group octadecylsilica C18 column [16,30,34]. The most common HPLC eluent contains a mixture of distilled water:methanol with a slow percentage of acetic acid run isocratically. Except for the technique of Carreto et al. [37], there is no isolation and identification MAA protocol able to completely isolate large MAAs mixtures on marine organisms. The method used by Carreto et al. [37] combines a C18 column with an acidic eluent. Although this approach is able to separate high mixtures of MAAs, it is a time-consuming analysis that we recommend only in case of samples with a complex mix of MAAs.

We have analyzed the 20% aqueous methanol and distilled water MAA extracts, using a Luna-C8 and a Poroshell-C18 columns. The characteristics of them are different; while the C8 have 5 µm particle size and a dimension of 4.06 mm x 250 mm, the C18 column have 4 µm particle size and a dimension of 3 mm x 250 mm. Therefore, besides the different material of each column, we have different particles sizes and thickness. Thus, this is why we had to decrease the flow using C18 from 0.5 mL/min to 0.3 mL/min. Recently, Rosic et al. [34] described a general method for the isolation and characterization of MAA compounds from red algae and symbiotic dinoflagellates using methanolic extracts and HPLC and LC-MS with electrospray ionization source interface. They used a C18 column (1.7 μm particle size) and concluded that porphyra-334, palythine, and mycosporine-glycine could not be separated*,* as these mixed MAAs appeared only in an elution peak at the beginning of the chromatogram. According to the results obtained in our work, the C18 column offered the worst results in terms of MAA separation using these seven procedures, although the particle sizes of the columns were different. However, Karsten et al. [28], who used three reverse-phase C8 and C18 HPLC columns, obtained better results than us using the Synergi C18 column with the same particle size and dimensions, but acquired from different companies, Phenomenex versus Agilent. They compared, similarly to this work, pure methanol, distilled water, and HPLC eluent as re-dissolution solvent for dried *Prasiola* and *Porphyra* extracts (*Pyropia* former *Porphyra*) [28]. On the contrary, Karsten et al. [28] found analytical problems using the C8 columns, this was not the case for this study; in fact, we used the same C8 Luna column, and we obtained better results than with the C18 column. One explanation to the mixed MAAs could be related to other highly polar, low molecular weight UV-absorbing substances unrelated to MAAs that are generally present in extracts of marine organisms. These molecules may interact with the eluent solvent and MAAs presents in the samples increasing their molecular weights and changing their polarity, and therefore their elution times [30]. Matrix effect is minimized in analysis of purified and partially purified MAA extracts [20,38]. Although, why this matrix effect could occur under some conditions and not others must be investigated.

According to our results, the highest MAA concentration was obtained by injecting the sample directly in HPLC after its extraction, regardless if it is extracted in distilled water or 20% methanol. The drying and subsequent re-dissolution of the pellets in the different studied solvents declined total MAA concentrations, as occurred in Karsten et al. [28]. With the independence of solvent of extraction, the MAA concentration when the sample was injected directly into the HPLC was approximately double compared to after drying the sample and resuspending it in 20% methanol. Consequently, methanol should be avoided as a re-dissolution solvent for the HPLC analysis in terms of estimation of total MAA concentration. However, these treatments (M4–W4), together with W1, led to improved peak separations. Although, the use of methanol as a re-dissolution solvent provokes double peaks with similar absorption maxima and, therefore, limits its use for qualitative MAA analysis. This result could also be related to matrix effect [39].

Other authors had studied MAA composition and concentration in the same species included in this work. Roleda et al. [40] found, in *A. vermiculophyllum* (former *Gracilaria vermiculophylla*), two MAAs but they were not identified. Lalegerie et al. [14] found eight different MAAs in the same species, some of them unidentified, but no total MAA concentration was shown. Barceló-Villalobos et al. [32] identified four MAAs, the same as in this work, porphyra-334, shinorine, palythine, and asterina-330. However, total MAA concentration of samples collected from the field was at least two times higher than our MAA concentration. Regarding *Crassiphycus corneus* (formerly *Hydropuntia* or *Gracilaria cornea*), Sinha et al. [41] identified shinorine and porphyra-334, but they did not show total MAA concentration. Figueroa et al. [42] found shinorine, pophyra-334, and palythine. The concentration in this species was very similar to our work. Álvarez-Gómez et al. [31] found four MAAs, but the concentration was much lower than those presented in this work. In another article, similar concentration and composition to that in our work were found [43,44], around 1 mg g^−1^, and porphyra-334, shinorine, palythine, asterina-330, and palythinol. MAAs in *G. longissima* were analyzed firstly by Torres et al. [45], but they identified asterina-330, palythinol, palythene, and usujirene. However, similar concentration and composition (porphyra-334, shinorine, palythine, asterina-330, and palythinol, and a concentration around 1 mg g^−1^) have been shown in this species by other authors [31,43,44]. Finally, concentration and composition of *Porphyra* species previously published did not differ from the results presented here. This species has porphyra-334 as the dominant MAA, and in minor concentrations, shinorine, palythine, asterina-330, and palythinol. It belongs to the Order Bangiales, which has the highest MAA concentration [17,46,47]. To our knowledge, it is the first time in the literature in which myricetin, a flavonoid found in several foods, has been detected in one species of the Family Gracilariaceae. Other flavonoids have been found in this taxonomic group, such as camptothecin and quercetin [48]. More studies are needed to quantify myricetin in this species and investigate the regulation of its synthesis. This flavonoid has shown anti-photoaging effects through its antioxidant and anti-inflammatory properties [49,50]. The beneficial effects have encouraged researchers to develop photo-protective products from natural sources [51]. It is known that onions, kale, lettuce, tomatoes, apples, grapes, berries, tea, and red wine are rich sources of flavonols [52]. However, it is not deeply studied in algae. A recent study has described the presence of myricetin, among other flavonoids, in *Caulerpa* spp. [53]. The last study revealed that *Caulerpa* spp. are a promising functional food ingredient and could be explored as daily dietary supplements. Higher content of flavonoids in seaweeds were reported [54]. In contrast, the flavonoid content of some of them was reported to be very low [55]. 

This study allows us to obtain a general procedure for MAA analysis in species with similar MAA profiles than those included in this study, i.e., those containing porphyra-334, shinorine, palythine, asterina-330, and palythinol. In fact, these are the most abundant MAAs in macroalgae. Therefore, for a routine analysis of those MAAs, we suggest the following procedures: the best protocol to optimize MAA characterization is the extraction of MAAs in distilled water, followed by the extract dryness, and the re-dissolution in pure methanol. However, in order to obtain a better estimation of the total MAA concentration, the extraction in distilled water and direct injection of the extract in HPLC should be used.

Our results entail important implications regarding the use of red macroalgae as promising candidates as environment-friendly sources of industrially important compounds, like MAAs, because of their photoautotrophic properties, which can convert solar energy and carbon dioxide into useful chemicals as it has been reported by Navarro et al. [56]. MAAs, in addition to protecting against erythema, can reduce other negative biological effects as photocarcinogenesis, immunosupression CHYS, photisomerization of urocanic acid, or photo-aging [11,25]. Additionally, the genus *Gracilaria* has high potential as a source of high-value compounds and extracts for several applications [57]. However, only agar is commercially exploited. A better use of the biomass from commercial cultivation of species from the Family *Gracilariaceae* may be an important alternative for exploiting the other bioactive components, such as MAAs and flavonoids. Further studies must be carried out.

## 4. Materials and Methods 

### 4.1. Biological Material

Specimens of *Agarophyton vermiculophyllum* (Ohmi) Gurgel, J.N.Norris et Fredericq were collected at Ria de Aveiro (40°38′N, 8°43′W), Portugal. *Crassiphycus corneus* (J.Agardh) Gurgel, J.N.Norris & Fredericq was collected from cultures in tanks located in a greenhouse of Gran Canaria, Spain (27°59′28′′N; 15°22′8′′W)*. Gracilariopsis longissima* (S.G.Gmelin) M.Steentoft, L.M.Irvine & W.F.Farnham were collected on River San Pedro (36°32’52” N; 6°12’33”W), Tarifa, Cádiz, Spain. *Pyropia leucosticta* (Thuret) Neefus & J.Brodie were collected on rocky shores from Lagos (36°28′N, 4°1′W), Málaga, Spain. Algae were dried by silica gel and stored under controlled humidity. Dried algal material was crushed to powder prior to extraction to guarantee homogeneity.

### 4.2. Extraction and Identification of MAAs

Analysis of MAAs was assayed according to Korbee-Peinado et al. [17] with some modifications. For the MAA extraction were used two different solvents: 20% aqueous methanol (*v*/*v*) and distilled water. Samples of dried algal (20 mg DW) were extracted for 2 h in screw-capped centrifuge vials filled with 1 mL 20% aqueous methanol (*v*/*v*) or distilled water in a waterbath at 45 °C. After this, 700 µL of the supernatant was evaporated to dryness under vacuum (Jouan evaporator centrifuge, France), except two samples of each species whose extracts in 20% aqueous methanol or distilled water were passed through a 0.2 µm membrane filter to be analyzed directly by HPLC. All samples were extracted in triplicate. In Figure 1, the seven protocols used in this work are represented. Three replicates were analyzed directly by HPLC (protocols with number 1). The other extracts were dried and re-dissolved in 700 µL 100% aqueous methanol (protocols with number 4), HPLC eluent (1.5% methanol (*v*/*v*) plus 0.15% acetic acid (*v*/*v*) in distilled water) (number 3) or distilled water (number 2), and mixed for 30 s. After passing through a 0.2 µm membrane filter, samples were analyzed with a Waters 600 HPLC system (Waters Cromatografía, Barcelona, Spain). Sample volumes of 20 µL were injected into two columns: a reverse-phase Luna C8 column (5 µm particle size; 4.06 mm × 250 mm, Phenomenex, Aschaffenburg, Germany) with a pre-column (C8, Octyl, MOS; Phenomenex) and an Infinity Lab Poroshell 120 reverse-phase C18 column (4 µm particle size; 3 mm × 250 mm, Agilent, Santa Clara, CA, USA). The mobile phase used as eluent was 1.5% methanol (*v*/*v*) plus 0.15% acetic acid (*v*/*v*) in distilled water, run isocratically at 0.5 mL min^−1^ with the Luna column and 0.3 mL min^−1^ with the Agilent column. The pressure was very high with higher fluxes in the last column. 

MAAs were detected online with a Waters Photodiode Array Detector 996 at 330 nm, and absorption spectra (290–400 nm) were recorded each second directly on the HPLC-separate peaks. Identification of MAAs was performed by comparison of the absorption spectra and retention times and by co-chromatography using high purified grade MAAs extracts (porphyra-334, shinorine, palythine y asterina-330) provided from the Unit of Photobiology of the Central Service for Research Support (SCAI, University of Málaga, Málaga, Spain). Quantification was made by using published extinction coefficients [58,59] and the formula found in Pliego-Cortes et al. [60]. When we obtained a mixed unidentifiable peak, we estimated the total MAA concentration using an average extinction coefficient for those MAAs present in the species [38].

The qualitative analysis was complemented by electrospray ionization mass spectrometry (ESI-MS) with a high-resolution mass spectrometer (model Orbitrap Q-Exactive, Thermo Scientific S.L., Bremen, Germany) provided with an electrospray ionization heated probe (HESI-II), in the premises of the Central Service for Research Support (SCAI, University of Málaga, Málaga, Spain). The mass spectrometer indicated 70–700 m/z in positive mode for MAA and negative mode for myricetin with a voltage of 3.5 kV

### 4.3. Statistical Analysis

Values of mean concentration and their standard deviations per treatment and column were calculated from three replicates. Total MAA concentration was tested by one-way analysis of variance (ANOVA) for each species. Cochran′s test was used to check the homogeneity of variances. A post-hoc Student Newman–Keuls (SNK) multiple comparison test was applied and significant differences are indicated with different letters in Figure 4. Analysis were done using the program STATISTICA (8.0, Dell and StatSoft, CA, USA).

## Figures and Tables

**Table 1:** Mycosporine-like amino acid composition, relative percentage (%), and mass spectrometry characterization for each analyzed species. AV: *Agarophyton vermiculophyllum*. CC: *Crassiphycus corneus*. GC: *Gracilariopsis longissima*. PL: *Pyropia leucosticta.*

Species	MAAs	% Relative	Mol. Formula	λ_max_ (nm)	Exact (ppm)	Calculated (*m/z* [M + H]^+^)	Observed (*m/z* [M + H]^+^)
	Porphyra-334	40.19 ± 1.33	C_14_H_22_N_2_O_8_	334	0.8	347.14489	347.14365
	Shinorine	10.63 ± 2.71	C_13_H_20_N_2_O_8_	334	1.4	333.12924	333.12799
**AV**	Palythine	42.61 ± 3.09	C_10_H_16_N_2_O_5_	320	2.9	245.11320	245.11247
	Asterina-330	4.97 ± 0.57	C_12_H_20_N_2_O_6_	330	1.8	289.13941	289.13840
	Porphyra-334	18.04 ± 3.85	C_14_H_22_N_2_O_8_	334	0.8	347.14489	347.14316
	Shinorine	49.20 ± 2.80	C_13_H_20_N_2_O_8_	334	1.4	333.12924	333.12781
**CC**	Palythine-serine	6.12 ± 1.42	C_11_H_18_N_2_O_6_	320	3.8	275.12376	275. 12271
	Asterina-330	1.27 ± 0.41	C_12_H_20_N_2_O_6_	330	1.8	289.13941	289.13840
	Palythinol	30.28 ± 1.74	C_13_H_22_N_2_O_6_	332	0.9	303.15506	303.15399
	Porphyra-334	5.36 ± 3.18	C_14_H_22_N_2_O_8_	334	0.8	347.14489	347.14371
	Shinorine	37.05 ± 9.79	C_13_H_20_N_2_O_8_	334	1.4	333.12924	333.12805
**GL**	Palythine	1.70 ± 0.20	C_10_H_16_N_2_O_5_	320	1.2	245.11320	245.11290
	Asterina-330	1.63 ± 0.45	C_12_H_20_N_2_O_6_	330	1.8	289.13941	289.13849
	Palythinol	59.28 ± 9.52	C_13_H_22_N_2_O_6_	332	0.9	303.15506	303.15421
	Porphyra-334	79.83 ± 1.98	C_14_H_22_N_2_O_8_	334	0.8	347.14489	347.14343
	Shinorine	3.77 ± 0.99	C_13_H_20_N_2_O_8_	334	1.4	333.12924	333.12799
**PL**	Palythine	6.26 ± 0.95	C_10_H_16_N_2_O_5_	320	1.2	245.11320	245.11227
	Asterina-330	7.69 ± 0.80	C_12_H_20_N_2_O_6_	330	1.8	289.13941	289.13821
	Palythinol	2.44 ± 0.16	C_13_H_22_N_2_O_6_	332	0.9	303.15506	303.15411

**Table 2:** Mycosporine-like amino acids (MAAs) identified in each analyzed species and protocols for their identification by HPLC using the C8-Luna column.

*Agarophyton vermiculophyllum*							
MAAs	Protocols						
	M1	M2	M3	M4	W1	W3	W4
Porphyra-334	•	•	•	•	•	•	•
Shinorine	•	•	•	•	•	•	•
Palythine	•	•	•	•	•	•	•
Asterina-330	•	•	•	•	•	•	•
*Crassiphycus corneus*							
	M1	M2	M3	M4	W1	W3	W4
Porphyra-334	•	•	•	•	•	•	•
Shinorine				•			•
Palythine-serine	•	•	•	•	•	•	•
Asterina-330	•	•	•	•	•	•	•
Palythinol				•			•
*Gracilariopsis longissima*							
	M1	M2	M3	M4	W1	W3	W4
Porphyra-334	•	•	•	•	•	•	•
Shinorine				•			•
Palythine	•	•	•	•	•	•	•
Asterina-330	•	•	•	•	•	•	•
Palythinol				•			•
*Pyropia leucosticta*							
	M1	M2	M3	M4	W1	W3	W4
Porphyra-334	•	•	•	•	•	•	•
Shinorine	•			•			•
Palythine	•	•	•	•	•	•	•
Asterina-330	•	•	•	•	•	•	•
Palythinol	•			•			•

**Table 3:** List of protocols indicating extraction and re-dissolution solvents, abbreviation, strengths, and weaknesses of each of them for Luna-C8 column.

Extraction Solvent	Re-dissolution Solvent	Abbreviation	Strengths	Weaknesses
20% Methanol	Direct HPLC analysis without re-dissolution	M1	High MAA concentration	Accumulation of impurities in the column; Toxic for cosmetic use
	Distilled water	M2	High MAA concentration	Some unidentified MAAs; Toxic for cosmetic use
	HPLC eluent	M3	Intermediate MAA concentration	Some unidentified MAAs; Toxic for cosmetic use
	100% Methanol	M4	Improved MAA separation	Low MAA concentration; Toxic for cosmetic use
Distilled water	Direct HPLC analysis without re-dissolution	W1	High MAA concentration; Suitable for cosmetic	Accumulation of impurities in the column
	HPLC eluent	W3	Intermediate MAA concentration;Suitable for cosmetic	Some unidentified MAAs
	100% Methanol	W4	Improved MAA separation; Suitable for cosmetic	Low MAA concentration

## Supplementary Materials

The following are available online at https://www.mdpi.com/1660-3397/18/1/27/s1. Figure S1: Mass spectrum of A (porphyra-334), B (shinorine), C (palythine), D (asterina-330), and E (myricetin) in *Agarophyton vermiculophylum.* Figure S2: Mass spectrum of A (porphyra-334), B (shinorine), C (palythine-serine), D (asterina-330), and E (palythinol) in *Crassiphycus corneus.* Figure S3: Mass spectrum of A (porphyra-334), B (shinorine), C (asterina-330), and D (palythinol) in *Gracilariopsis longissima.* Figure S4: Mass spectrum of A (porphyra-334), B (shinorine), C (palythine), and D (asterina-330) in *Pyropia leucosticta.* Figure S5: HPLC normalized chromatograms of MAAs identified in *Agarophyton vermyculophyllum* by seven methodological protocols using the Luna-C8 column. Figure S6: HPLC normalized chromatograms of MAAs identified in *Agarophyton vermyculophyllum* by seven methodological protocols using the Infinity Lab Poroshell 120 C18 column. Figure S7. HPLC normalized chromatograms of MAAs identified in *Crassiphycus corneus* by seven methodological protocols using the Luna-C8 column. Figure S8. HPLC normalized chromatograms of MAAs identified in *Crassiphycus corneus* by seven methodological protocols using the Infinity Lab Poroshell 120 C18 column. Figure S9. HPLC normalized chromatograms of MAAs identified in *Gracilariopsis longissima* by seven methodological protocols using the Luna-C8 column. Figure S10. HPLC normalized chromatograms of MAAs identified in *Gracilariopsis longissima* by seven methodological protocols using the Infinity Lab Poroshell 120 C18 column.
