# Supplementary material for: Quantitative and Qualitative HPLC Analysis of Mycosporine-Like Amino Acids Extracted in Distilled Water for Cosmetical Uses in Four Rhodophyta
Source: Mar Drugs. 2019 Dec 28;18(1):27. doi: 10.3390/md18010027 (PMC7024326; doi:10.3390/md18010027)
Supplement: Supplementary file 1 [file marinedrugs-18-00027-s001.pdf]

marine drugs

# Supplementary materials

# Quantitative and Qualitative HPLC Analysis of Mycosporine-Like Amino Acids Extracted in Distilled Water for Cosmetical Uses in Four Rhodophyta

Patricia Chaves-Peña [1], Francisca de la Coba [2], Felix L. Figueroa [1] and Nathalie Korbee [1,*]

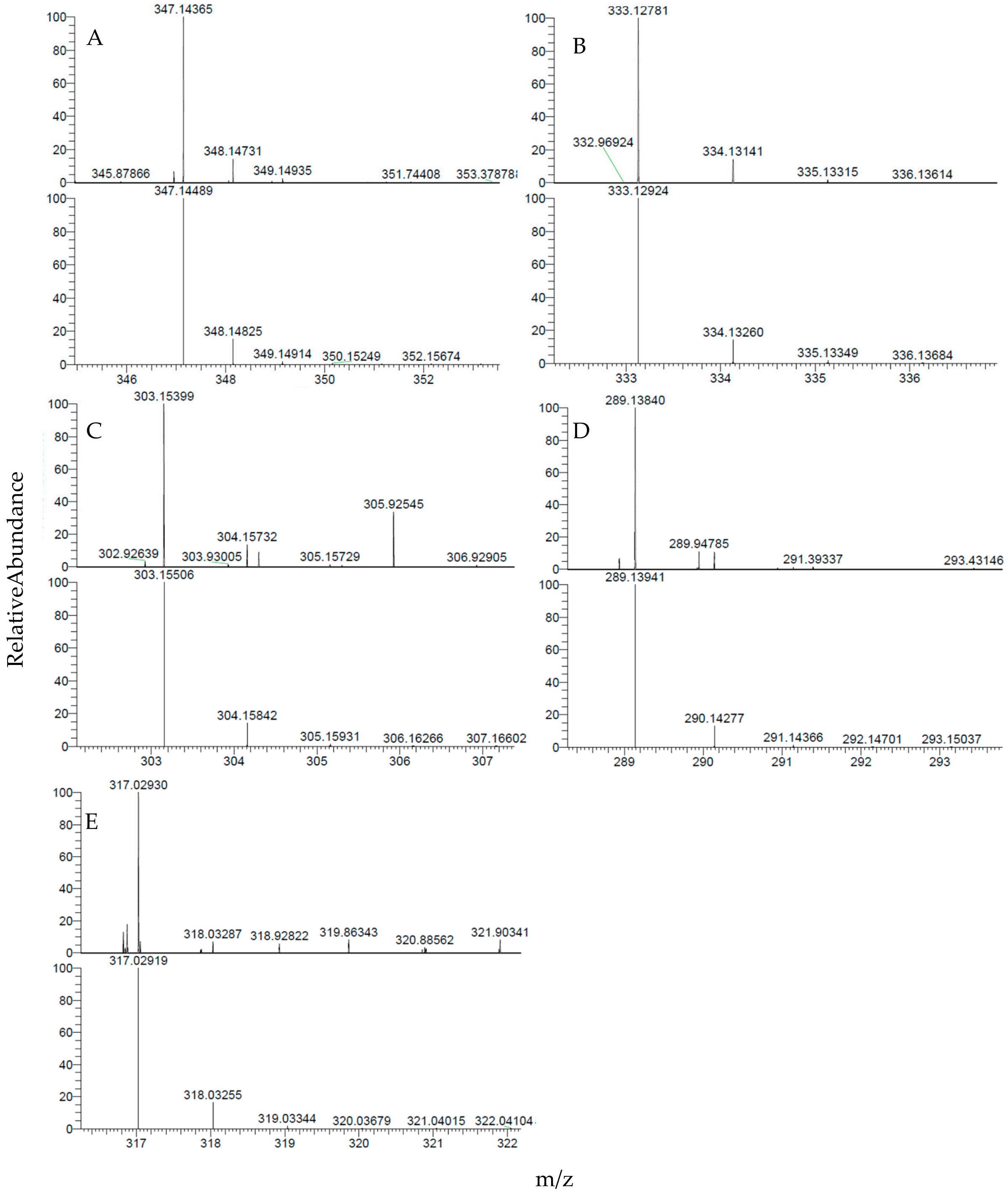


**Figure S1.** Mass spectrum of A (porphyra-334), B (shinorine), C (palythine), D (asterina-330) and E (myricetin) in *Agarophyton vermiculophyllum*.

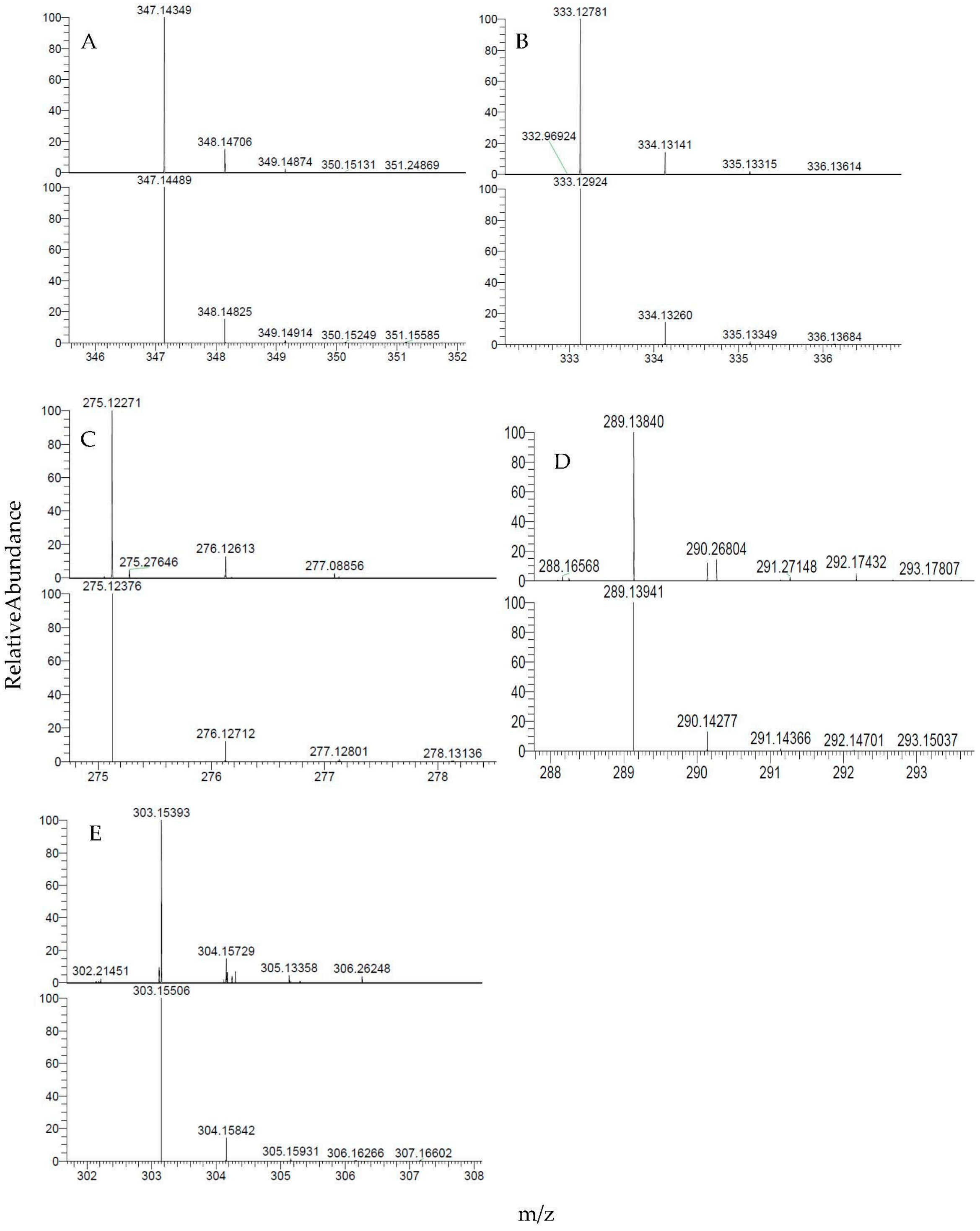


**Figure S2.** Mass spectrum of A (porphyra-334), B (shinorine), C (palythine-serine), D (asterina-330) and E (palythinol) in *Crassiphycus corneus*.

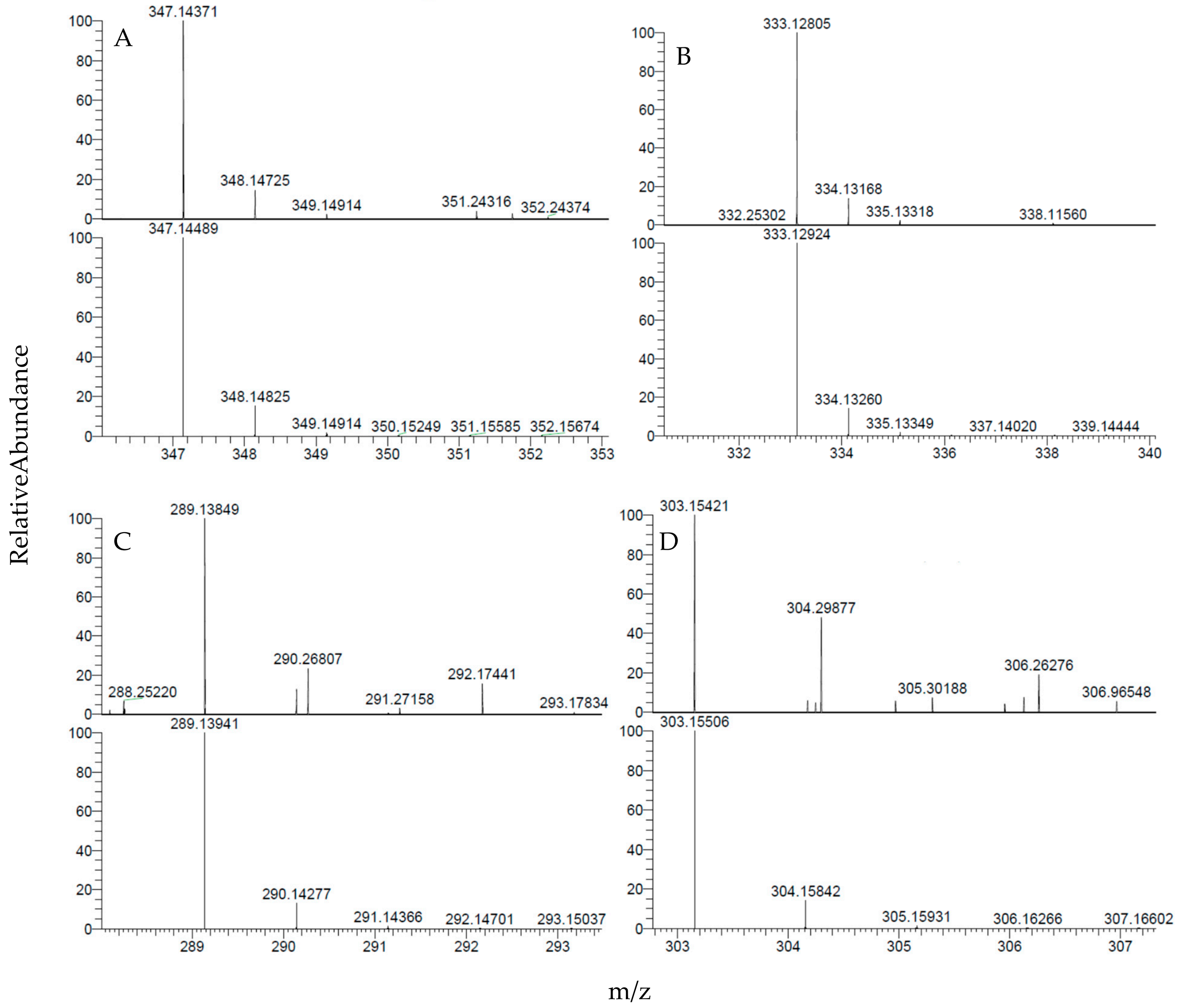


**Figure S3.** Mass spectrum of A (porphyra-334), B (shinorine), C (asterina-330) and D (palythinol) in *Gracilariopsis longissima*.

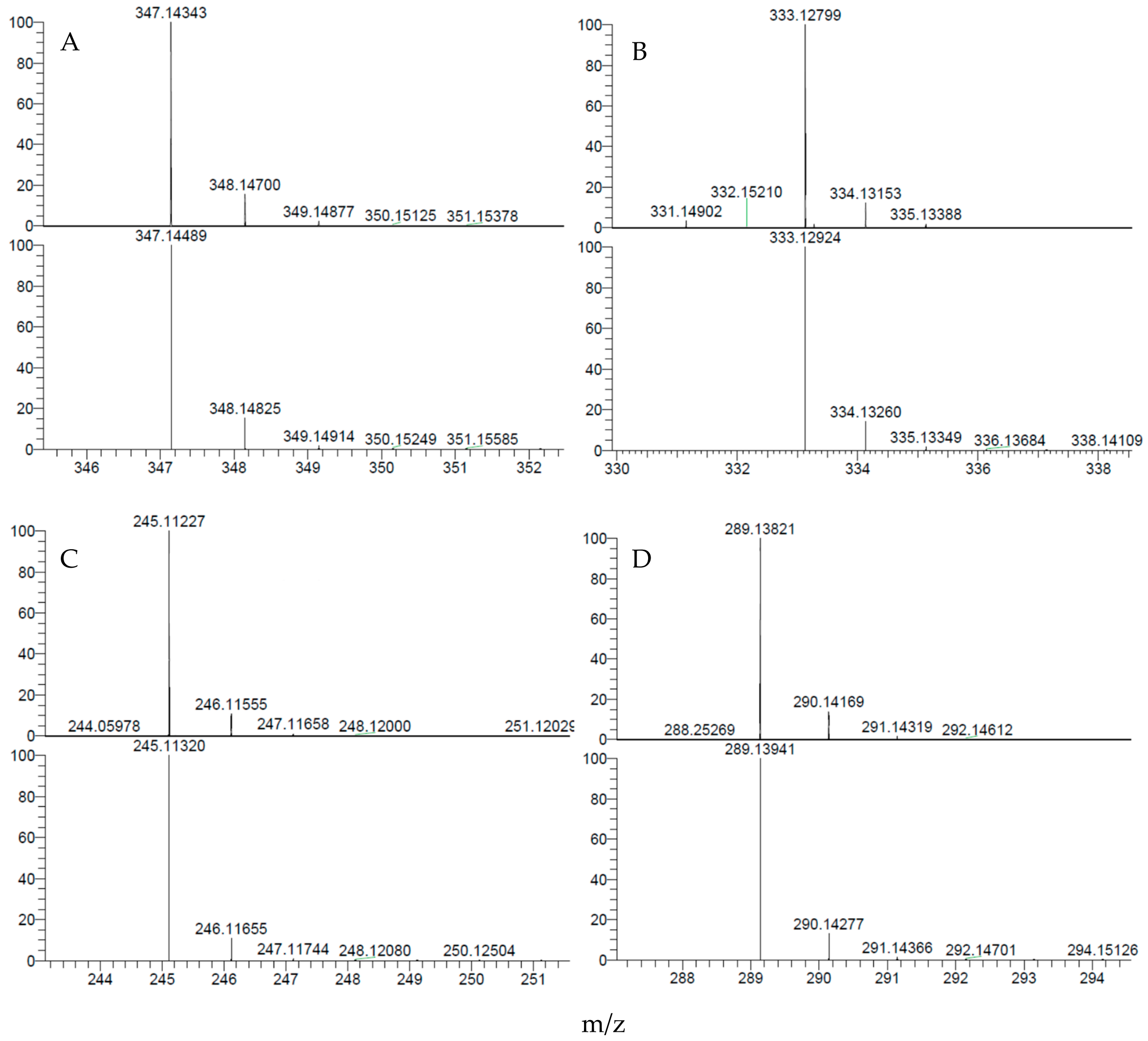


**Figure S4.** Mass spectrum of A (porphyra-334), B (shinorine), C (palythine) and D (asterina-330) in *Pyropia leucosticta*.

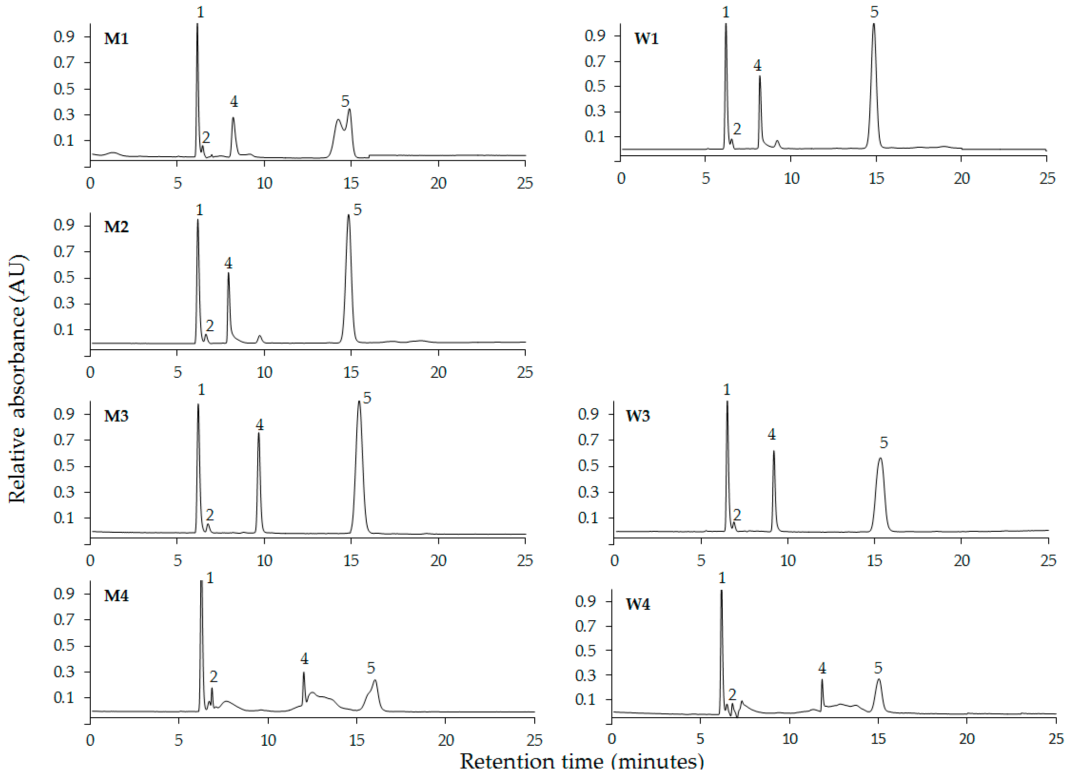


**Figure S5.** HPLC normalized chromatograms of MAAs identified in *Agarophyton vermiculophyllum* by seven methodological protocols using the Luna-C8 column. The code of protocol is indicated based on extraction and re-dissolution solvents used (see figure 1 legend). Numbers indicate: 1 (palythine), 2 (asterina-330), 4 (shinorine), and 5 (porphyra-334).

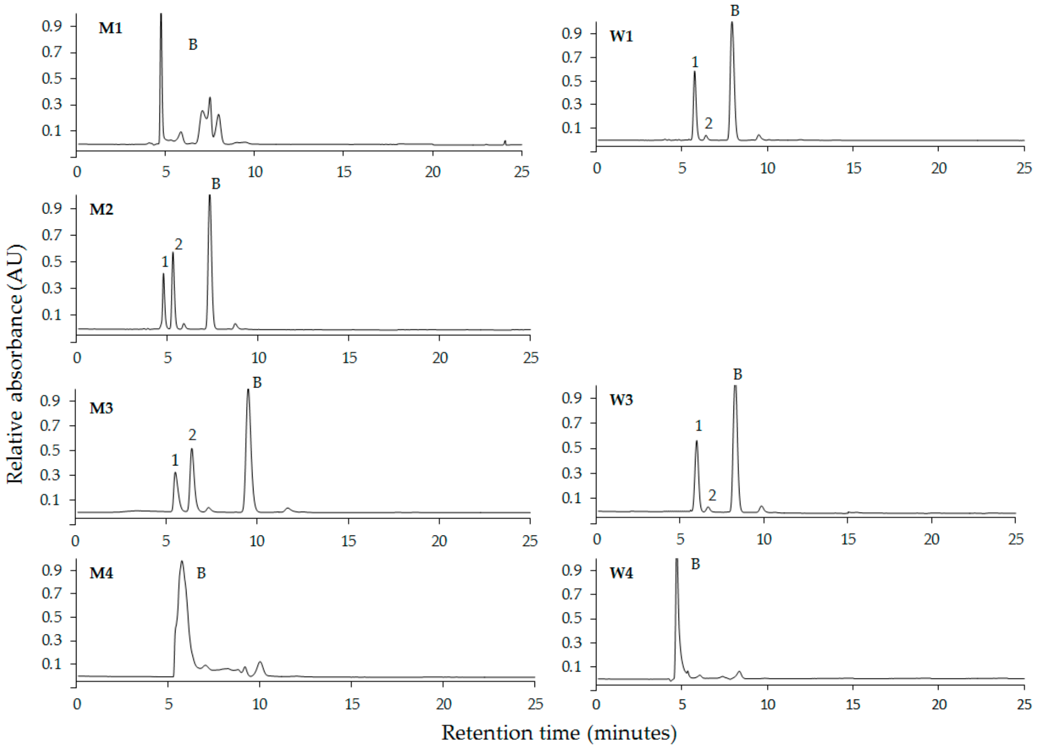


**Figure S6.** HPLC normalized chromatograms of MAAs identified in *Agarophyton vermiculophyllum* by seven methodological protocols using the Infinity Lab Poroshell 120 C18column. The code of protocol is indicated based on extraction and re-dissolution solvents used (see figure 1 legend). Numbers indicate: 1 (palythine), 2 (asterina-330), and B (mixed unidentifiable peaks).

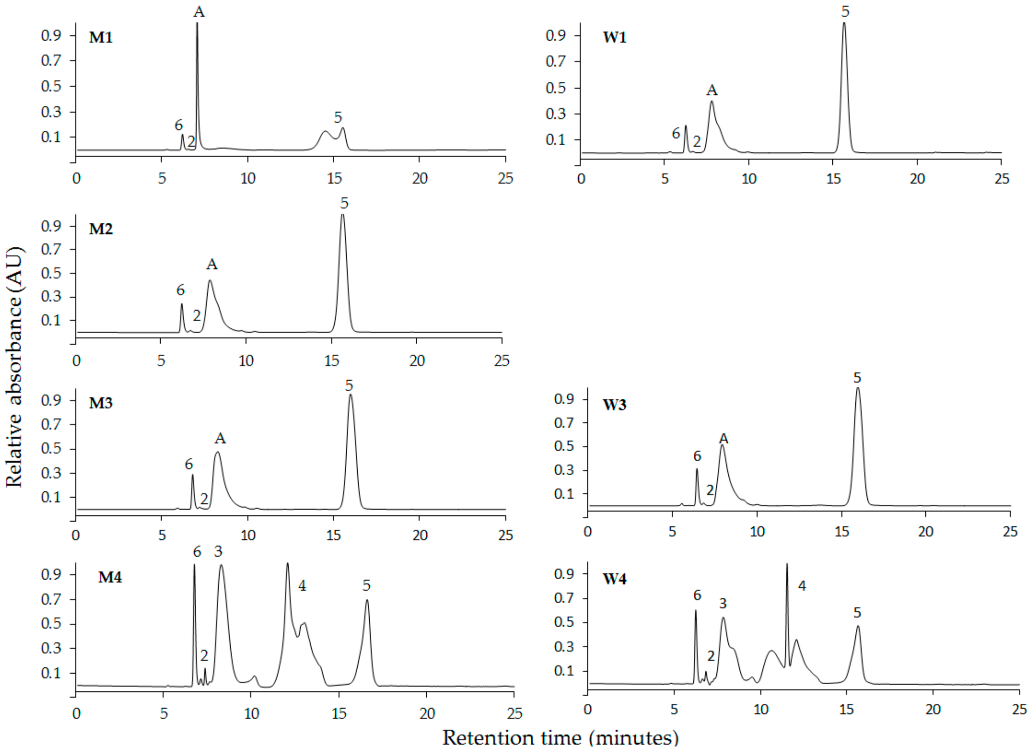


**Figure S7.** HPLC normalized chromatograms of MAAs identified in *Crassiphycus corneus* by seven methodological protocols using theLuna-C8 column. The code of protocol is indicated based on extraction and re-dissolution solvents used (see figure 1 legend). Numbers indicate: 2 (asterina-330), 3 (palythinol), 4 (shinorine), 5 (porphyra-334), 6 (palythine-serine), and A (mixture of palythinol and shinorine).

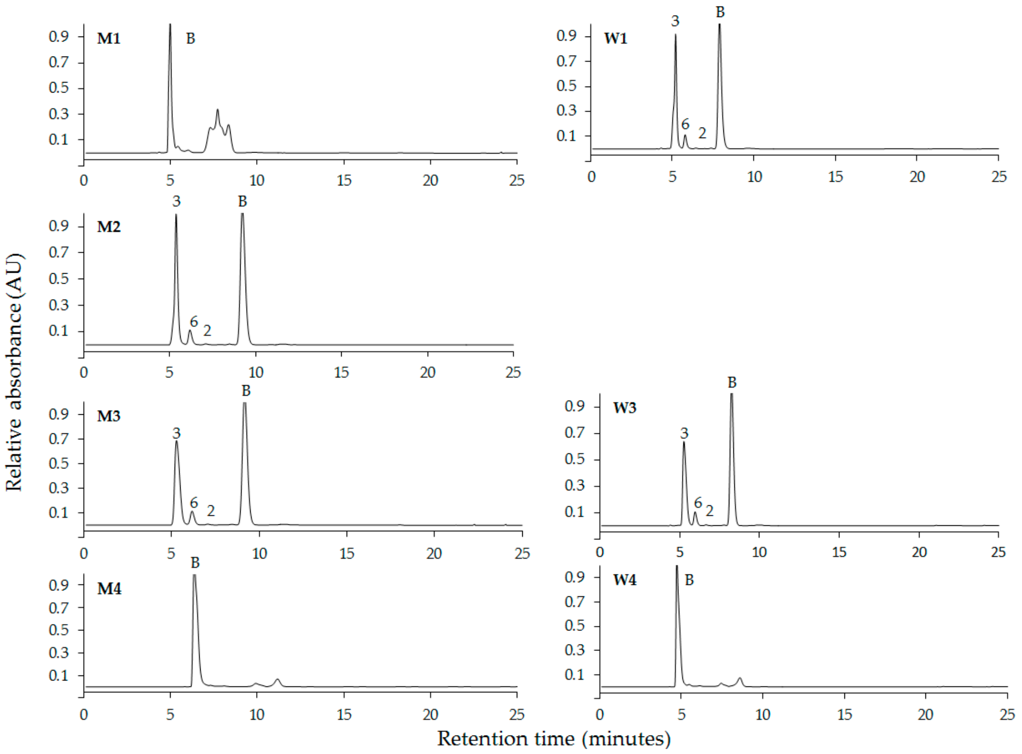


**Figure S8.** HPLC normalized chromatograms of MAAs identified in *Crassiphycus corneus* by seven methodological protocols using the Infinity Lab Poroshell 120 C18 column. The code of protocol is indicated based on extraction and re-dissolution solvents used (see figure 1 legend). Numbers indicate: 2 (asterina-330), 3 (palythinol), 6 (palythine-serine), and B (mixed unidentifiable peaks).

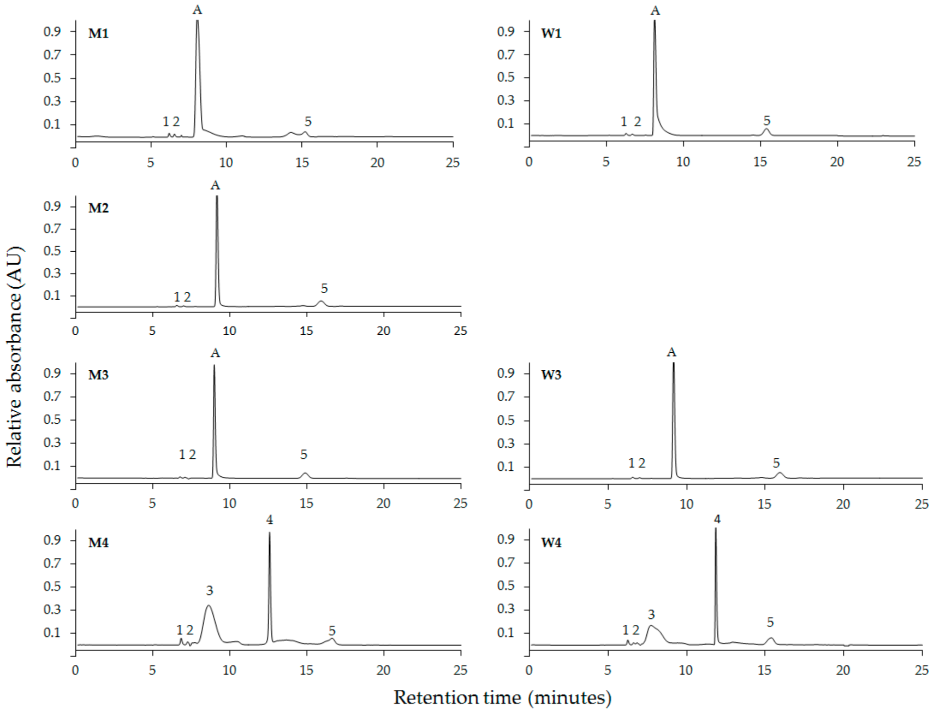


**Figure S9.** HPLC normalized chromatograms of MAAs identified in *Gracilariopsis longissima* by seven methodological protocols using the Luna-C8 column. The code of protocol is indicated based on extraction and re-dissolution solvents used (see figure 1 legend). Numbers indicate: 1 (palythine), 2 (asterina-330), 3 (palythinol), 4 (shinorine), 5 (porphyra-334), and A (mixture of palythinol and shinorine).

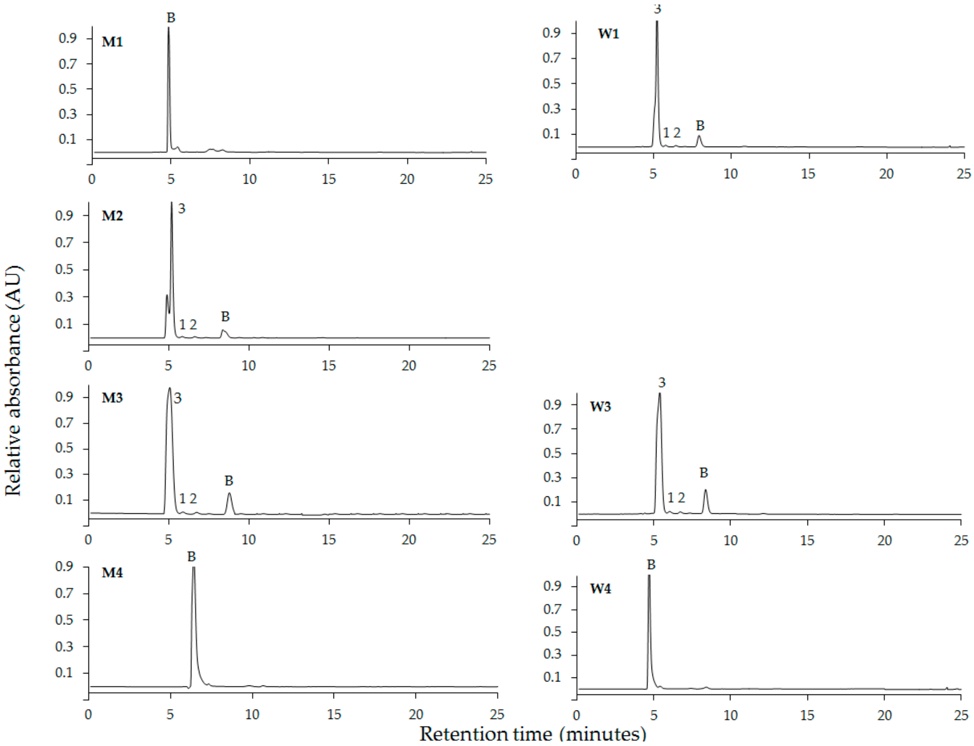


**Figure S10.** HPLC normalized chromatograms of MAAs identified in *Gracilariopsis longissima* by seven methodological protocols using the Infinity Lab Poroshell 120 C18 column. The code of protocol is indicated based on extraction and re-dissolution solvents used (see figure 1 legend). Numbers indicate: 1 (palythine), 2 (asterina-330), 3 (palythinol), and B (mixed unidentifiable peaks).
